# Mechanisms of force depression caused by different types of physical exercise studied by direct electrical stimulation of human quadriceps muscle

**DOI:** 10.1007/s00421-016-3473-0

**Published:** 2016-09-16

**Authors:** Albertas Skurvydas, Gediminas Mamkus, Sigitas Kamandulis, Vilma Dudoniene, Dovile Valanciene, Håkan Westerblad

**Affiliations:** 1 Institute of Sports Science and Innovation, Lithuanian Sports University, Lithuania, Sporto 6, 44221 Kaunas, Lithuania; 2 Department of Physiology and Pharmacology, Karolinska Institutet, 171 77 Stockholm, Sweden

**Keywords:** Prolonged low-frequency force depression, Recovery, Isometric contraction, Wingate cycling, Eccentric contractions

## Abstract

**Purpose:**

Force production frequently remains depressed for several hours or even days after various types of strenuous physical exercise. We hypothesized that the pattern of force changes during the first hour after exercise can be used to reveal muscular mechanisms likely to underlie the decline in muscle performance during exercise as well as factors involved in the triggering the prolonged force depression after exercise.

**Methods:**

Nine groups of recreationally active male volunteers performed one of the following types of exercise: single prolonged or repeated short maximum voluntary contractions (MVCs); single or repeated all-out cycling bouts; repeated drop jumps. The isometric force of the right quadriceps muscle was measured during stimulation with brief 20 and 100 Hz trains of electrical pulses given before and at regular intervals for 60 min after exercise.

**Results:**

All exercises resulted in a prolonged force depression, which was more marked at 20 Hz than at 100 Hz. Short-lasting (≤2 min) MVC and all-out cycling exercises showed an initial force recovery (peak after ~ 5 min) followed by a secondary force depression. The repeated drop jumps, which involve eccentric contractions, resulted in a stable force depression with the 20 Hz force being markedly more decreased after 100 than 10 jumps.

**Conclusions:**

In accordance with our hypothesis, the results propose at least three different mechanisms that influence force production after exercise: (1) a transiently recovering process followed by (2) a prolonged force depression after metabolically demanding exercise, and (3) a stable force depression after mechanically demanding contractions.

## Introduction

Our ability to perform precise movements depends on the fine-tuned activation of skeletal muscles. The force output of skeletal muscle in vivo is controlled by the number of activated motor units and their discharge frequency. In addition, the force output at a given activation level can be modified by alterations within the muscle fibers. Intense physical activity generally induces intramuscular alterations that tend to decrease force production (Allen et al. 2008). However, there are also situations where force is potentiated, for instance, a phenomenon known as post-activation potentiation where twitch force and rate of force development are increased following a brief tetanic contraction (Sweeney et al. 1993). Moreover, processes causing force reduction and potentiation can coexist, which makes it difficult to assess the relative importance of different processes (Fowles and Green 2003; Rassier and Macintosh 2000; Skurvydas and Zachovajevas 1998).

Factors causing changes in muscle fiber force production can act dynamically and alter force within seconds, or they can be more long-lasting and require hour(s) or even days to be reversed. A long-lasting force depression frequently observed after various types of physical exercise is more marked at low than at high stimulation frequencies and hence referred to as “prolonged low-frequency force depression” (PLFFD) (Allen et al. 2008). Edwards et al. (1977) originally referred to this long-lasting force decrease as low-frequency fatigue, but this term has since been used to describe numerous different conditions and, therefore, lost its precision. The force-frequency relationship is non-linear with an initial steep part at low frequencies (~10–30 Hz in human muscle) and saturation at higher frequencies (>50 Hz) (Edwards et al. 1977; Merton 1954). During human voluntary contractions individual motor units usually fire at frequencies on the steep part of the force-frequency relationship (Grimby and Hannerz 1977; Marsden et al. 1971). Thus, PLFFD will have a large impact on the neuronal programming of contractions and increase subjects’ perceived effort to achieve a given force output (Carson et al. 2002).

PLFFD was first described by Edwards and co-workers (1977), who studied the recovery after severe fatigue induced by repeated voluntary contraction of human adductor pollicis muscles under ischemic conditions. Subsequently PLFFD has been observed after various types of metabolically demanding contractions both in vivo and in vitro (Allen et al. 2008; Keeton and Binder-Macleod 2006; Place et al. 2010). A decreased force production in muscle fibers can, in principle, be due to (1) reduced free myoplasmic Ca^2+^ concentration ([Ca^2+^]_i_) during contraction, (2) decreased myofibrillar Ca^2+^ sensitivity, and (3) reduced ability of contractile machinery to produce force (Westerblad and Allen 1996). On a simplified model, factors (1) and (2) would result in a larger force depression at low than at high stimulation frequencies due to the sigmoidal shape of the force-[Ca^2+^]_i_ relationship, whereas factor (3) would give a similar force decrease at all stimulation frequencies (Allen et al. 2008; Bruton et al. 2008).

A particularly prominent type of PLFFD is observed after unaccustomed eccentric contractions, which impose large mechanical stress on muscle (Dargeviciute et al. 2013; Davies and White 1981; Newham et al. 1987). This force depression is accompanied by signs of general muscle damage, such as, delayed muscle soreness, swelling, protein leakage and inflammation (Clarkson and Hubal 2002), as well as specific myofibrillar disorganization and disrupted sarcomeres (Fridén et al. 1983; Proske and Morgan 2001; Yu et al. 2004). Thus, myofibrillar disturbances induced by mechanically demanding eccentric contractions is another factor that can cause a larger force depression at low than at high stimulation frequencies.

In the present study, nine groups of recreationally active young men each performed one type of physical exercise that previously has been shown to induce PLFFD: repeated or prolonged isometric contractions continued until exhaustion or a severe force reduction (Edwards et al. 1977; Jones et al. 1982); repeated concentric contractions including repeated bouts of all-out Wingate cycling (Hill et al. 2001; Place et al. 2015); repeated eccentric contractions including repeated drop jumps (Dargeviciute et al. 2013; Davies and White 1981; Newham et al. 1987; Skurvydas et al. 2011). We used the following general exercise protocols: (1) prolonged or repeated isometric maximum voluntary contractions (MVCs), which may involve gradually developing activation failure and require anaerobic metabolism since blood flow is occluded; (2) brief bouts of metabolically demanding all-out cycling; (3) repeated drop jumps, which involve eccentric contractions and impose large mechanical but little metabolic stress on muscles. The isometric force induced by brief 20 and 100 Hz trains of electrical pulses given to the right quadriceps muscle was measured before and after exercise. The rationale behind using electrical stimulation is that it allowed us to assess changes in muscle function independent of neuronal activation. Forces were followed for 1 h after exercise and we hypothesized that the pattern of force changes during this first hour would reveal mechanisms likely to underlie the decline in muscle performance during exercise as well as factors involved in the triggering PLFFD.

## Methods

### Subjects

The study involved nine groups of male volunteers and their characteristics are displayed in Table 1. All subjects were physically active and participated in recreational activities 2–3 times per week. They were asked to refrain from any exercise for 1 week prior to the experiment. Each group performed only one of the exercise protocols described below to avoid problems with results being affected by previous exercise; for instance, a single bout of eccentric contractions induces a protection against deleterious effects of subsequent eccentric contractions (repeated bout effect) and this protection lasts for several weeks (e.g. Nosaka et al. 2005). The study was approved by the Kaunas Regional Ethics Committee and is consistent with the principles outlined in the Declaration of Helsinki. Each subject read and signed a written, informed consent form prior to participation.

**Table 1:** Characteristics of subjects (mean ± SEM)

Intervention	*N*	Age (years)	Weight (kg)	Height (m)	Pt (Nm)	HRT (ms)	P20 (Nm)	P100 (Nm)
30 s MVC	12	20.5 ± 0.7	80.4 ± 1.2	1.82 ± 0.03	17.2 ± 2.1	65.5 ± 3.2	100.2 ± 6.1	188.2 ± 9.3
60 s MVC	14	20.8 ± 0.3	81.1 ± 0.6	1.84 ± 0.01	17.5 ± 2.2	68.4 ± 2.4	107.5 ± 6.2	183.4 ± 7.3
120 s MVC	12	19.9 ± 0.4	80.8 ± 1.3	1.79 ± 0.04	16.1 ± 2.1	64.9 ± 2.9	102.5 ± 5.4	184.2 ± 8.6
12 × 5 s MVC	13	21.2 ± 0.9	79.2 ± 0.9	1.78 ± 0.02	16.8 ± 1.9	64.5 ± 2.8	101.5 ± 5.2	186.2 ± 7.9
30 s Win	12	22.5 ± 0.7	83.1 ± 1.6	1.85 ± 0.03	17.1 ± 2.5	67.4 ± 2.6	104.4 ± 6.3	178.4 ± 11.4
3 × 30 s Win	12	24.5 ± 0.9	77.9 ± 1.6	1.80 ± 0.03	17.9 ± 2.2	67.7 ± 3.1	103.5 ± 8.3	184.9 ± 11.5
12 × 5 s all-out cycling	14	23.1 ± 0.9	79.9 ± 1.0	1.81 ± 0.01	16.5 ± 2.4	69.4 ± 2.6	108.5 ± 8.9	181.4 ± 7.8
10 DJ	12	20.8 ± 0.4	80.1 ± 0.7	1.79 ± 0.02	16.9 ± 2.9	64.4 ± 3.1	104.5 ± 10.2	174.4 ± 8.6
100 DJ	14	21.5 ± 0.6	77.7 ± 1.2	1.82 ± 0.02	15.1 ± 2.1	64.9 ± 2.7	101.5 ± 7.5	178.9 ± 10.5

*MVC* isometric maximum voluntary contraction, *Win* Wingate cycling, *DJ* drop jumps, *Pt* peak twitch force, *HRT* twitch half-relaxation time, *P20 and P100* peak force with 20 and 100 Hz stimulation, respectively

### Exercise protocols

#### Isometric MVC

Four different protocols were used: 30 s MVC; 60 s MVC; 120 s MVC; 12 × 5 s MVCs at 60 s interval. We used single MVCs of different durations (30–120 s) to assess how the duration of continuous activation affects force production after exercise. Continuous MVC causes fatigue that involves decreased activation from the central nervous system and/or impaired sarcolemmal excitability (Bigland-Ritchie et al. 1983; Kent-Braun 1999); to minimize such decreases in activation, we also used repeated short (5 s) MVCs. Subject sat upright in the dynamometer chair (System 3; Biodex Medical Systems, Shiley, New York, USA) with a vertical back support. Isometric knee extension was performed with the knee joint positioned at an angle of 60° (0°—full knee extension). The subjects were verbally encouraged to exert and maintain maximal force during the contractions.

#### All-out cycling

Three different all-out cycling protocols were used: one bout of 30 s Wingate cycling (1 × 30 s Win) (Bar-Or 1987); three bouts of 30 s Wingate cycling at 4 min interval (3 × 30 s Win); twelve bouts of 5 s all-out cycling at 1 min interval (12 × 5 s all-out). In addition to one bout of Wingate cycling, we used three bouts to study the effect of repeating this highly exhaustive type of dynamic exercise on force production after exercise. We also used 12 × 5 s all-out cycling where, in contrast to 30 s all-out Wingate cycling, power output was expected to remain high during the cycling bouts and hence the effect of a higher average power exercise could be assessed. The subjects were seated on a mechanically braked cycle ergometer (Monark, 824E, Sweden) equipped with a basket weight loading system and appropriate adjustments were made to ensure an optimal riding position. After a full acceleration without resistance to reach the maximal pedal revolution rate, a brake weight corresponding to 7.5 % (approximated to the nearest 0.1 kg) of the subject’s weight was applied to initiate the all-out cycling. The power output was averaged over 5 s periods.

#### Drop jumps

Subjects performed either 10 or 100 drop jumps at 30 s interval; the long interval was chosen to minimize the metabolic stress. This type of exercise involves mechanically demanding eccentric contractions and the two different protocols were used to assess whether detrimental effects on force production require only a few (10 drop jumps) or many (100 drop jumps) of eccentric contractions. Drop jumps were performed from a height of 0.5 m. Upon landing, knee bending was counteracted by eccentric knee extensor contraction until 90° knee angle was reached, immediately followed by a maximal concentric rebound contraction (Kamandulis et al. 2010). The knee angle at the end of the deceleration phase was visually controlled by an experienced researcher and subjects were immediately instructed to modify the way the jumps were performed if the angle diverged from the intended 90°. Subjects landed on a contact mat (Powertimer Testing System, Newtest, Oulu, Finland), which allowed immediate feed-back of jumping heights and subjects were encouraged to execute each rebound jump as high as possible.

### Direct electrical muscle stimulation and force measurements

Electrically evoked peak isometric force of the right leg quadriceps muscle was measured with a Biodex isokinetic dynamometer. The subjects sat upright with the knee joint positioned at an angle of 60°. Shank, trunk, and shoulders were stabilized by belts. Direct electrical muscle stimulation was applied using two carbonized rubber electrodes covered with a thin layer of electrode gel (ECG–EEG Gel; Medigel, Modi’in, Israel). One of the electrodes (6 cm × 11 cm) was placed transversely across the width of the proximal portion of the quadriceps muscle next to the inguinal ligament; the other electrode (6 cm × 20 cm) covered the distal portion of the muscle above the patella. An electrical stimulator (MG 440; Medicor, Budapest, Hungary) delivered square-wave pulses of 1 ms duration. Each subject was familiarized to the experimental procedures and electrical stimulation on a separate occasion before the actual testing. The amplitude of the square-wave current pulses required to obtain maximum force was determined by gradually increasing the voltage until no increment in force response was elicited by a 10 % voltage increase. We measured peak force during 1 s trains of current pulses given at 20 or 100 Hz and separated by a 5 s resting period. Each frequency was only tested once at each time point. Tests were performed before and directly, 5, 10, 30 and 60 min after exercise. The test directly after exercise was performed ~30 s after the end of isometric MVCs, and ~2 min after the end of all-out cycling and drop jumps where subjects had to move to the dynamometer chair. Peak MVC force was measured before and 5, 10, 30 and 60 min after exercise.

The reliability of force measurements for voluntary and electrically evoked contractions was tested on two consecutive days and under the same conditions in physically active male volunteers (*n* = 19). The coefficient of variation (the ratio of the standard deviation to the mean) for MVC, 100 Hz and 20 Hz evoked force was 2.5 % (3.7 Nm), 4.9 % (6.2 Nm), and 5.8 % (4.5 Nm), respectively.

We used direct electrical muscle stimulation to be able to assess exercise-induced changes in muscle function independent of any changes neuronal muscle activation. Several studies have shown major differences in the response to exercise performed with voluntary contractions vs. similar exercise with electrically evoked contractions (e.g. Hansen et al. 2009; Jubeau et al. 2008). In the present study, all types of exercise were performed with voluntary contractions and only the testing before and after exercise involved contractions induced by direct electrical stimulation. Thus, it appears unlikely that the observed changes in force production after exercise were significantly affected by the brief electrical stimulation.

### Statistical analysis

Data are presented as mean ± SEM. One way analysis of variance (ANOVA) for repeated measures was used to determine statistical differences in force production during recovery vs. before exercise. If significant effects were found, post hoc testing was performed with Bonferroni correction for multiple comparisons. The level of significance was set at 0.05.

## Results

### MVC—isometric contractions

**Fig. 1:**
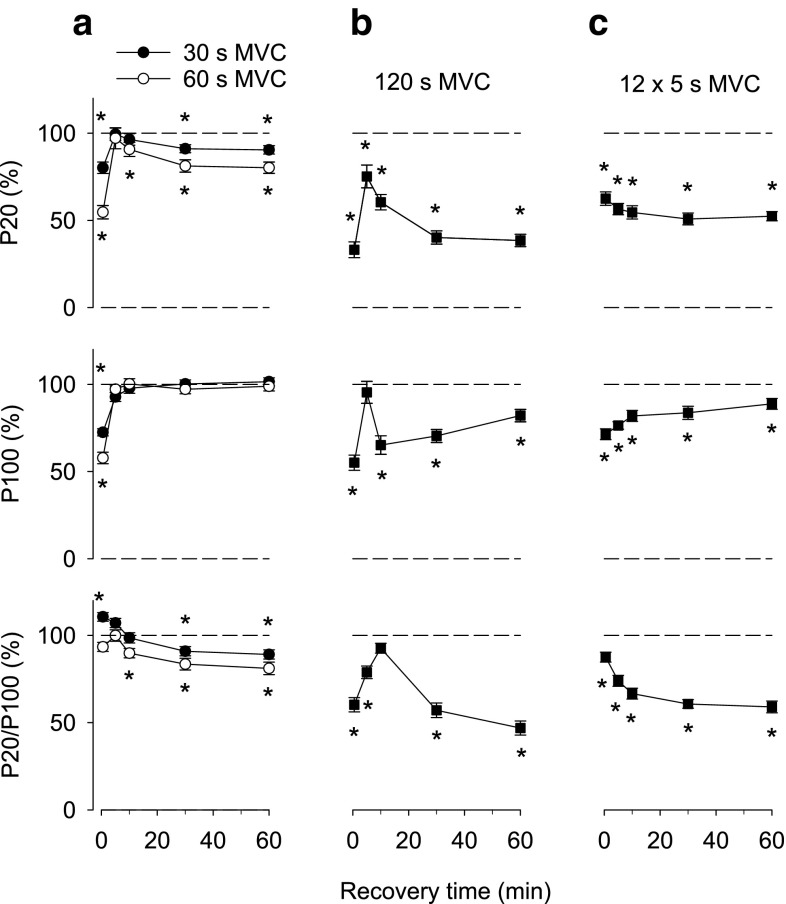
Electrically induced isometric force production at 20 Hz (P20) and 100 Hz (P100) of supramaximal stimulation, and the 20/100 Hz force ratio presented relative to the baseline value in each subject. Data are mean (±SEM; *n* = 12–14, see Table 1) and were obtained ~30 s, and 5, 10, 30 and 60 min after: **a** 30 and 60 s MVC; **b** 120 s MVC; **c** 12 × 5 s MVC. *P < 0.05 vs. before MVC contractions; in **a** *asterisk* above and below data points refer to 30 and 60 s MVC, respectively

Initial experiments were performed with continuous MVCs. Directly after the fatiguing contractions, 20 and 100 Hz forces were decreased by ~25 % with 30 s MVCs and by ~45 % with 60 s MVCs (Fig. 1a). Forces then showed an initial full recovery, which was followed by a secondary decrease at 20 Hz whereas the 100 Hz force remained high. Thus, there was a prolonged decrease of 20 Hz force and the 20/100 Hz force ratio of ~10 and ~20 % after the 30 and 60 s MVCs, respectively.

The force decrease was more severe when the MVC duration was increased to 120 s, but the general pattern during recovery was similar to that with the shorter MVCs; that is, an early improvement followed by a prolonged decrease, which now also involved 100 Hz force (Fig. 1b). At 60 min of recovery, 20 and 100 Hz force and the 20/100 Hz force ratio were decreased by about 60, 20 and 50 %, respectively.

The force produced during a prolonged MVC gradually declines due to fatigue development, which involves decreased activation from the central nervous system and/or impaired sarcolemmal excitability (Bigland-Ritchie et al. 1983; Kent-Braun 1999), both of which will limit the energy metabolic stress during the contraction. Having this in mind, we also tested the effect of repeated short MVCs (12 × 5 s contractions at 60 s interval), which would induce less activation failure and hence put a larger stress on energy metabolism. Despite a total duration of contraction of only 60 s, the force–time integral of these repeated contractions was similar to that of the 120 s MVCs and consequently the average force was about twice as large with the repeated MVCs (Table 2). The magnitude of the prolonged force depression after the repeated MVCs was similar to that observed with 120 s MVCs, but the initial transient improvement did not occur (Fig. 1c).

**Table 2:** Force measurements during either continuous (30–120 s) or repeated brief (12 × 5 s) maximum voluntary contractions (mean ± SEM)

Duration (s)	Number of subjects	Force–time integral (kNm × s)	Average force (Nm)
30	12	6.75 ± 0.12	225.0 ± 3.8
60	14	9.90 ± 0.21	165.0 ± 3.5
120	12	14.10 ± 0.39	118.0 ± 3.3
12 × 5	13	14.10 ± 0.56	235.0 ± 9.3

### All-out cycling—dynamic contractions

**Fig. 2:**
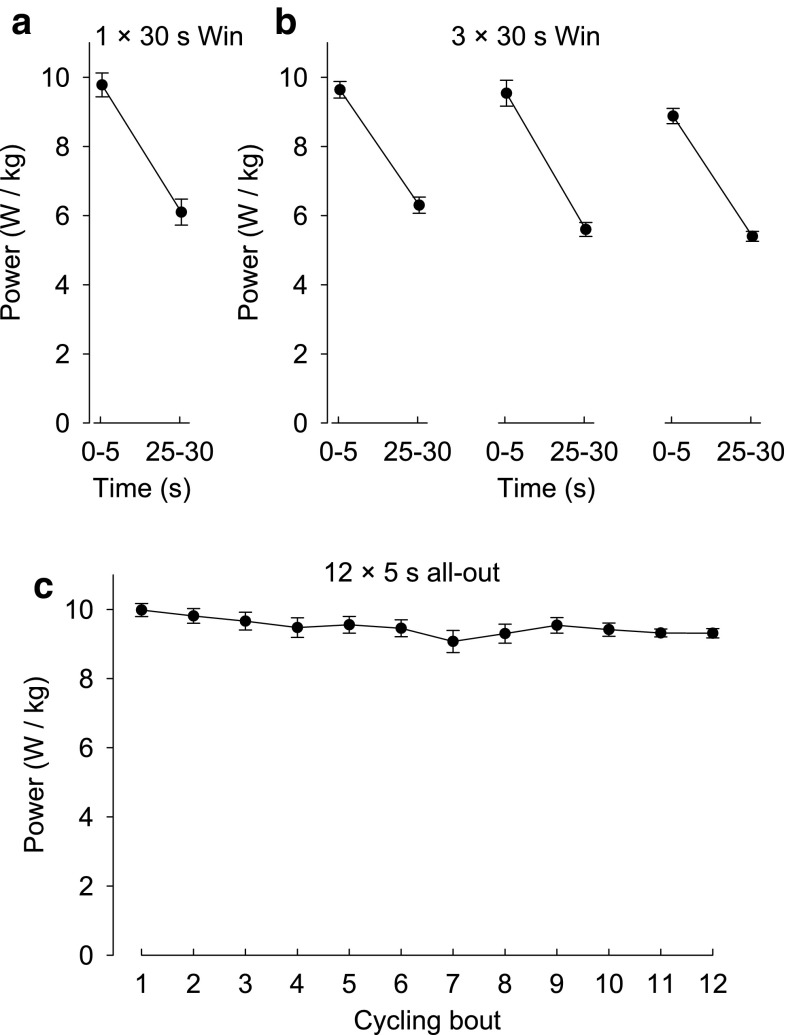
Power output during all-out cycling bouts. Data are mean (±SEM; *n* = 12–14, see Table 1) and were obtained during Wingate cycling, **a** 1 × 30 s and **b** 3 × 30 s at 4 min interval, and **c** during 12 × 5 s all-out cycling at 1 min interval. Averaged power during the first and last 5 s periods are for the 30 s cycling bouts

**Fig. 3:**
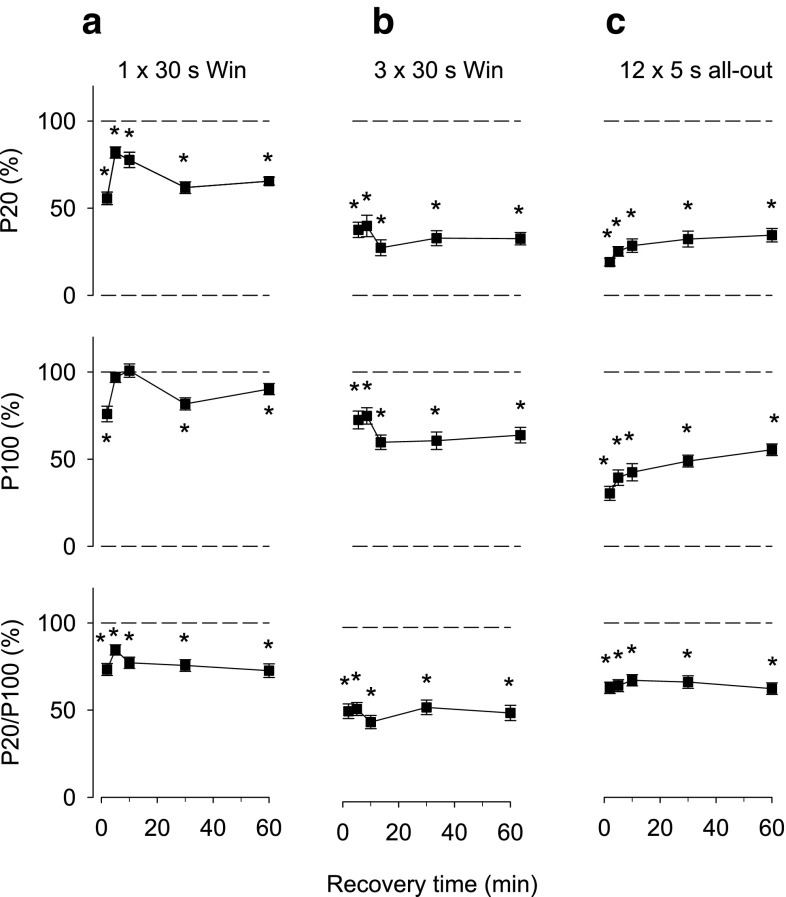
Electrically induced isometric force production at 20 Hz and 100 Hz of supramaximal stimulation, and the 20/100 Hz force ratio presented relative to the baseline value in each subject. Data are mean (±SEM; *n* = 12–14, see Table 1) and were obtained ~2, 5, 10, 30 and 60 min after Wingate cycling, **a** 1 × 30 s and **b** 3 × 30 s at 4 min interval, and **c** after 12 × 5 s all-out cycling at 1 min interval. *P < 0.05 vs. before cycling exercise

In the next series of experiments, subjects performed all-out cycling, which involves energetically highly demanding concentric contractions. Subjects performed either 30 s of all-out Wingate cycling (Bar-Or 1987) or bouts of 5 s all-out cycling. The power output showed a marked decline during each 30 s bout, whereas it was well maintained during the 5 s bouts (Fig. 2). The pattern of force changes after one bout of 30 s Wingate cycling was similar to that observed with the prolonged MVCs: an initial force improvement, which was followed by a prolonged secondary reduction especially of 20 Hz force (Fig. 3a). A more severe force depression was observed after three Wingate cycling bouts performed at 4 min interval (Fig. 3b). There was a slight tendency of an initial force recovery after the three Wingate cycling bouts and thereafter all measured parameters remained severely depressed. In a final set of cycling experiments, subjects performed 12 × 5 s all-out cycling bouts at 1 min interval. This resulted in a prolonged force depression of a magnitude similar to that observed with 3 × 30 s Wingate cycling, but it was not accompanied by any sign of transient early recovery (Fig. 3c).

### Drop jumps—eccentric contractions

**Fig. 4:**
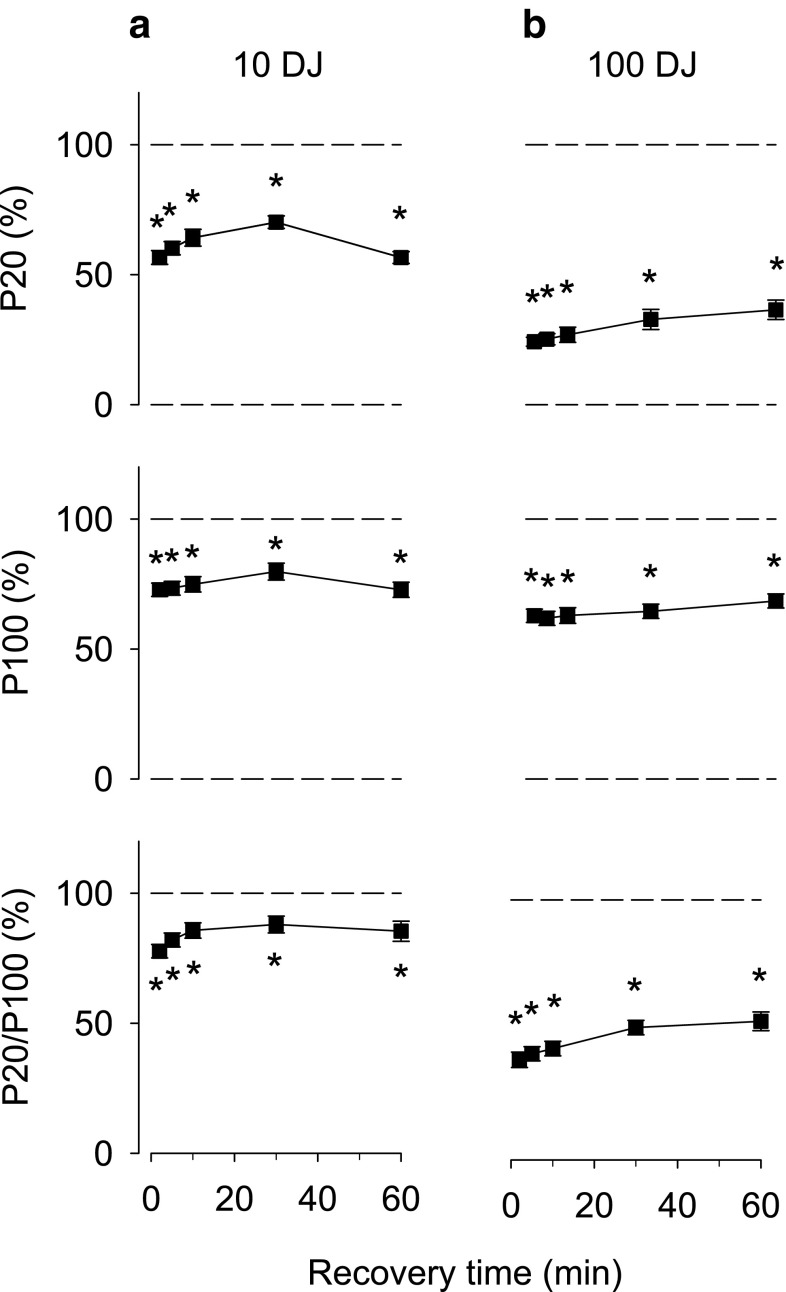
Electrically induced isometric force production at 20 Hz and 100 Hz of supramaximal stimulation, and the 20/100 Hz force ratio presented relative to the baseline value in each subject. Data are mean (±SEM; *n* = 12–14, see Table 1) and were obtained ~2, 5, 10, 30 and 60 min after **a** 10 or **b** 100 drop jumps performed at 30 s interval. *P < 0.05 vs. before drop jumps

In a final series experiments, subjects performed drop jumps at 30 s interval. This kind of exercise involves repeated eccentric contractions, which means that muscles are exposed to large mechanical stress whereas the demand on energy metabolism is limited. The force at 20 and 100 Hz stimulation and the 20/100 Hz force ratio were all markedly decreased directly after the 10 drop jumps and they remained depressed throughout the 60 min recovery period (Fig. 4a). More severe decreases were observed after the 100 drop jumps with reductions ranging from ~35 % for 100 Hz force to ~65 % for 20 Hz force (Fig. 4b).

### MVC forces after exercise

**Fig. 5:**
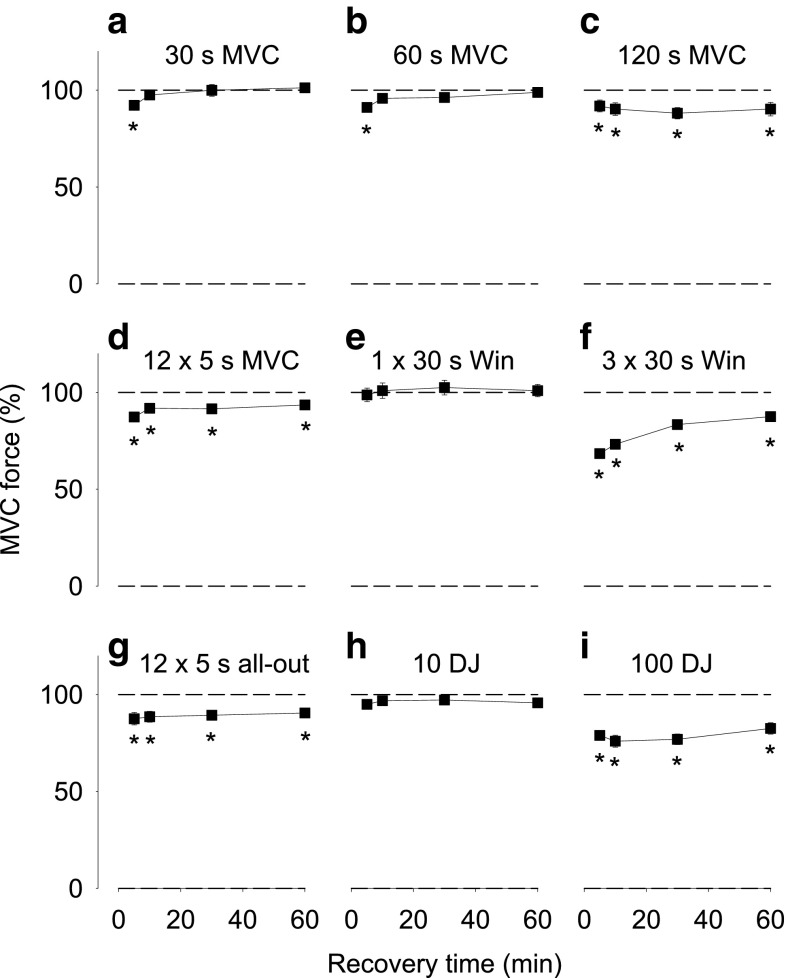
MVC forces after exercise presented relative to the baseline value obtained before exercise, which was set to 100 % in each subject. Data are mean (±SEM; *n* = 12–14, see Table 1) and were obtained 5, 10, 30 and 60 min after **a** 30 s MVC, **b** 60 s MVC, **c** 120 s MVC, **d** 12 × 5 s MVC, **e** 1 × 30 s and **f** 3 × 30 s Wingate cycling, **g** 12 × 5 s all-out cycling, **h** 10 and **i** 100 drop jumps. *P < 0.05 vs. before exercise

Maximum voluntary contraction forces were followed between 5 and 60 min after each of the above presented exercises (Fig. 5). The force reductions after exercise were generally smaller than those obtained with electrical muscle stimulation, which implies that the impaired muscular force production can be compensated for by increased neuronal activation.

## Discussion

This study was based on the hypothesis that the pattern of force changes during the first hour after different types exercise would reveal mechanisms likely to underlie the decline in muscle performance during exercise as well as factors involved in the triggering PLFFD. In accordance with this hypothesis, our results propose at least three different mechanisms that influence force production after exercise: (1) a fast process that develops during metabolically demanding exercise and is reversed within 10 min of recovery; (2) a slowly developing and prolonged process initiated by metabolically demanding exercise; (3) a prolonged process that develops during exercise with mechanically demanding eccentric contractions.

In this study we followed the force produced in response to direct electrical stimulation of quadriceps muscles after exercise. An advantage of the present approach with electrical muscle stimulation is that altered muscle function can be assessed without influence from any changes in the neuronal muscle activation. However, it must be noted that altered muscle activation by the central nervous system can also affect physical performance after exercise.

### Rapid initial recovery

A transient initial force recovery was observed with single MVCs (30–120 s duration) and with a single Wingate cycling bout (30 s duration). A common property of these exercises is a shorter duration from the start to the end of exercise (≤2 min) than with repeated MVCs (12 × 5 s with 60 s rest between contractions, totally 12 min) and the all-out cycling protocols (3 × 30 s at 4 min interval, totally 9.5 min; 12 × 5 s at 1 min interval, totally 12 min). Thus, these results indicate that the prolonged force depression (2) developed already during the more long-lasting exercises and hence these showed no fast recovery phase. Two likely mechanisms underlying the decreased force production after the single MVCs and the single Wingate cycling can be proposed. First, prolonged continuous contractions are accompanied by decreased central activation of motor units and possibly impaired action potential propagation within the muscle fibers (Bigland-Ritchie et al. 1983; Kent-Braun 1999). This type of activation failure can be rapidly reversed. For instance, reducing the stimulation frequency during ongoing, electrically induced contractions may actually increase force production, presumably by limiting K^+^- and Na^+^-fluxes over the sarcolemma and hence counteracting problems related to high-frequency action potential propagation (Jones et al. 1979; Westerblad et al. 1990). Second, a major metabolic factor causing decreased force production in acute fatigue is myoplasmic accumulation of inorganic phosphate ions due to breakdown of phosphocreatine (Allen et al. 2008; Dahlstedt et al. 2000). Phosphocreatine recovers to the control level, and hence the increase in inorganic phosphate ions vanishes, within a few minutes after prolonged contractions (Henriksson et al. 1986). To conclude, two mechanisms might explain the rapid initial recovery: reversal of deficient activation and the return of myoplasmic inorganic phosphate ions down to control levels. Deficient activation appears more likely to occur during the prolonged MVCs, where the requirement of a continuous generation of action potentials imposes a high risk of problems related to large K^+^- and Na^+^-fluxes (Bigland-Ritchie et al. 1983; Jones et al. 1979; Kent-Braun 1999; Westerblad et al. 1990). Conversely, Wingate cycling involves repeated brief contractions followed by rest periods, which means a lower risk of action potential failure, whereas the demand on rapid energy metabolism is high and large increases inorganic phosphate are likely to occur.

### Slowly developing, prolonged force depression initiated by metabolically demanding exercise

A prolonged force depression that was more prominent at 20 Hz than at 100 Hz stimulation was observed after all MVC and all-out cycling protocols. This type of PLFFD can theoretically be due to decreased sarcoplasmic reticulum (SR) Ca^2+^ release and/or decreased myofibrillar Ca^2+^ sensitivity (Allen et al. 2008). Recent studies aimed at identifying the mechanism(s) behind this force depression have revealed a key role of increased production of reactive oxygen/nitrogen species (ROS). The results from these studies show a coherent picture where the cellular ROS handling determines whether the dominating cause of the force depression is decreased SR Ca^2+^ release or reduced myofibrillar Ca^2+^ sensitivity (Cheng et al. 2016), Decreased SR Ca^2+^ release dominates in conditions where accumulation of superoxide anions (O_2_^·−^) is favored; conversely, reduced myofibrillar Ca^2+^ sensitivity is the dominating cause with facilitated clearance of O_2_^·−^, which can be achieved by increased endogenous concentration of superoxide dismutase (converts O_2_^·−^ to hydrogen peroxide (H_2_O_2_)) or application of exogenous antioxidants (Bruton et al. 2008; Cheng et al. 2015; Watanabe et al. 2015). The SR Ca^2+^ release channel, the ryanodine receptor 1 (RyR1), has been shown to be particularly sensitive to ROS-induced modifications resulting in impaired function and muscle weakness (Andersson et al. 2011; Bellinger et al. 2008). In a recent study (Place et al. 2015), we showed a marked PLFFD and a striking RyR1 fragmentation in muscles of recreationally active subjects after 6 × 30 s Wingate cycling bouts. Intriguingly, the same exercise caused a similar PLFFD in elite endurance athletes, but in this case the RyR1 remained intact and the difference can be explained by a higher superoxide dismutase expression in elite athletes (Place et al. 2015). Thus, the mechanism behind PLFFD induced by MVCs and Wingate cycling bouts can, depending on the training status, be either impaired SR Ca^2+^ release or reduced myofibrillar Ca^2+^ sensitivity.

### Prolonged force depression initiated by exercise with mechanically demanding eccentric contractions

Repeated drop jumps resulted in marked PLFFD, which was more severe after 100 than after 10 drop jumps and which tended to be more stable than after the metabolically more demanding types of exercise (MVC and all-out cycling). Thus, no initial transient recovery was observed even after 10 drop jumps where the total exercise duration was 4.5 min (9 × 30 s), i.e., less than the 5 min required for the initial force increase to reach its peak after short-lasting MVCs and Wingate cycling. Unaccustomed eccentric contractions are followed by signs of general muscle damage, such as, delayed onset muscle soreness, swelling, protein leakage and inflammation (Clarkson and Hubal 2002). Accordingly, we have previously shown that repeated drop jumps cause a marked PLFFD, which was not fully reversed even after 14 days (Dargeviciute et al. 2013; Skurvydas et al. 2011). They also induced severe delayed onset muscle soreness and substantially increased plasma creatine kinase activity, which indicates substantial protein leakage, and the magnitude and duration of these signs of muscle damage were larger after 100 than after 50 drop jumps (Dargeviciute et al. 2013; Skurvydas et al. 2011). Eccentric contractions exert a large mechanical stress on activated muscle fibers and a likely cause of the force depression after these contractions is myofibrillar damage and disorganized sarcomeres (Fridén et al. 1983; Proske and Morgan 2001; Yu et al. 2004). Still, muscle problems induced by eccentric contractions might also be linked to increased ROS production (Kerksick et al. 2010; Kon et al. 2007; Nikolaidis et al. 2008; Pal et al. 2013) and changes in cellular Ca^2+^ handling (Balnave and Allen 1995; Gehlert et al. 2012; Ingalls et al. 1998). If ROS- and/or Ca^2+^-dependent processes are involved in the PLFFD after drop jumps, these processes are unlikely to be identical to those underlying PLFFD after MVC and all-out cycling exercises, because they were triggered by markedly different major challenges (mechanical vs. metabolic stress) and they developed with different time-courses. Moreover, additional complex interactions may occur; for instance, delayed increases in ROS have been observed in skeletal muscle after eccentric contractions and these have been linked to leukocyte infiltration and inflammation (Nikolaidis et al. 2008).

## Conclusions

We followed force production induced by direct electrical stimulation of quadriceps muscles for 60 min after different types of physical exercise and distinguish three processes that affect force:

1. A transient initial force recovery that peaked ~ 5 min after short-lasting metabolically demanding exercise. This transient recovery is likely to be due to reversal of fatigue-induced activation failure and/or increase in myoplasmic inorganic phosphate ions.
2. A delayed, long-lasting force depression after metabolically demanding exercise that can be explained by ROS-dependent decreases in SR Ca^2+^ release and/or myofibrillar Ca^2+^ sensitivity.
3. A prolonged force depression developing during mechanically demanding eccentric contractions that is likely to be mainly due to myofibrillar disintegrity.

## Abbreviations

ANOVA: Analysis of variance
[Ca^2+^]_i_: Free myoplasmic Ca^2+^ concentration
DJ: Drop jumps
HRT: Twitch half-relaxation time
MVC: Maximum voluntary contraction
P20: Peak force with 20 Hz stimulation
P100: Peak force with 100 Hz stimulation
PLFFD: Prolonged low-frequency force depression
Pt: Peak twitch force
SR: Sarcoplasmic reticulum
ROS: Reactive oxygen/nitrogen species
RyR1: Ryanodine receptor 1
Win: Wingate cycling
